# Monomeric Immunoglobulin A from Plasma Inhibits Human Th17 Responses *In Vitro* Independent of FcαRI and DC-SIGN

**DOI:** 10.3389/fimmu.2017.00275

**Published:** 2017-03-14

**Authors:** Chaitrali Saha, Mrinmoy Das, Veerupaxagouda Patil, Emmanuel Stephen-Victor, Meenu Sharma, Sandra Wymann, Monika Jordi, Cédric Vonarburg, Srini V. Kaveri, Jagadeesh Bayry

**Affiliations:** ^1^Institut National de la Santé et de la Recherche Médicale, Paris, France; ^2^Sorbonne Universités, UPMC Univ Paris 06, Centre de Recherche des Cordeliers, Paris, France; ^3^Research Department, CSL Behring AG, Bern, Switzerland; ^4^Université Paris Descartes, Sorbonne Paris Cité, Paris, France

**Keywords:** monomeric IgA, Th17, IL-17, natural antibodies, IVIG, FcαRI, CD89, DC-SIGN, Treg

## Abstract

Circulating immunoglobulins including immunoglobulin G (IgG) and IgM play a critical role in the immune homeostasis by modulating functions of immune cells. These functions are mediated in part by natural antibodies. However, despite being second most abundant antibody in the circulation, the immunoregulatory function of IgA is relatively unexplored. As Th17 cells are the key mediators of a variety of autoimmune, inflammatory, and allergic diseases, we investigated the ability of monomeric IgA (mIgA) isolated from pooled plasma of healthy donors to modulate human Th17 cells. We show that mIgA inhibits differentiation and amplification of human Th17 cells and the production of their effector cytokine IL-17A. mIgA also suppresses IFN-γ responses under these experimental conditions. Suppressive effect of mIgA on Th17 responses is associated with reciprocal expansion of FoxP3-positive regulatory T cells. The effect of mIgA on Th17 cells is dependent on F(ab′)_2_ fragments and independent of FcαRI (CD89) and DC-SIGN. Mechanistically, the modulatory effect of mIgA on Th17 cells implicates suppression of phosphorylation of signal transducer and activator of transcription 3. Furthermore, mIgA binds to CD4^+^ T cells and recognizes in a dose-dependent manner the receptors for cytokines (IL-6Rα and IL-1RI) that mediate Th17 responses. Our findings thus reveal novel anti-inflammatory functions of IgA and suggest potential therapeutic utility of mIgA in autoimmune and inflammatory diseases that implicate Th17 cells.

## Introduction

CD4^+^ T-helper (Th) cells play an important role in the immune responses against both pathogens and self-antigens. Based on distinct cytokine and transcription factor profiles, several subsets of CD4^+^ Th cells have been elucidated. These include Th1, Th2, and CD4^+^CD25^+^ regulatory T cells (Tregs). In addition, Th17 cells that are characterized by lineage-specific transcription factor retinoic acid-related orphan receptor C (RORC) and the secretion of signature cytokine IL-17A are also identified (1). Phosphorylation of signal transducer and activator of transcription 3 (STAT3) is essential for the differentiation and amplification of Th17 cells. Th17 cells have been shown to play an important role in the defense against extracellular pathogens and in the pathogenesis of a variety of autoimmune, inflammatory, and allergic diseases (1, 2). Therefore, modulation of Th17 responses represents one of the fast evolving therapeutic options for the treatment of autoimmune and systemic inflammatory diseases.

Immunoglobulin A (IgA) is the most prevalent antibody at mucosal sites and the second most abundant antibody in the circulation. Secretory IgA at mucosal sites is dimeric in nature. It has been proposed that high affinity secretory IgA prevents mucosal colonization of invading pathogens and low affinity secretory IgA regulates commensal bacteria (3, 4). In the circulation, IgA is mostly monomeric although nearly 10–20% of circulating IgA are presented as dimeric or polymeric in nature (5). Currently, three bona fide receptors have been identified for IgA. FcαRI (CD89) expressed on various innate cells such as monocytes, macrophages, dendritic cells (DCs), and neutrophils is specific for IgA and mediates effector functions of IgA. The other two receptors, polymeric Ig receptor and Fcα/μR recognize both IgA and IgM and are therefore not specific for IgA. In addition to the aforementioned receptors, two alternative IgA receptors have also been identified. They are the asialoglycoprotein receptor that is implicated in the catabolism of IgA, and the transferrin receptor that mediates deposition of IgA in tissues (6).

Although triggering of FcαRI by IgA immune complexes or polymers induces activation of immune cells (7–9) *via* complete phosphorylation of tyrosine residues of immunoreceptor tyrosine-based activation motif (ITAM) within the associated FcRγ adaptors, naturally occurring monomeric IgA (mIgA) in the plasma was found to exert inhibitory effects on the activation of immune cells by triggering inhibitory ITAM (ITAMi) signaling through the associated FcRγ chain and recruitment of tyrosine phosphatase Src homology 2 domain-containing phosphatase-1 (SHP-1) (10–13). mIgA also induces death in activated neutrophils (14) and inhibits complement deposition mediated by anti-ganglioside antibodies (15). The anti-inflammatory effects of mIgA have been explored in various experimental models (10–13, 16).

Thus, so far anti-inflammatory effects of mIgA have been elucidated mainly in the context of innate immune cells and FcαRI. It is not known whether anti-inflammatory effects of mIgA observed in various experimental models are solely due to the modulation of innate cells or also due to anti-inflammatory effects on the cells of adaptive immune compartment and particularly CD4^+^ T cells that are critical players in the pathogenesis of autoimmune and inflammatory diseases. Therefore, in view of emerging roles of Th17 cells in the pathogenesis of autoimmune, allergy, and inflammatory diseases, we explored the immunomodulatory role of mIgA isolated from the pooled plasma of healthy donors on the human Th17 cell differentiation, amplification, and secretion of effector cytokine IL-17A. Our data indicate that mIgA binds to CD4^+^ T cells independent of FcαRI (CD89), and reciprocally regulates human Th17 and FoxP3-positive Treg cells. The effect of mIgA on Th17 cells is dependent on F(ab′)_2_ fragments and implicates suppression of phosphorylation of STAT3. Our data thus reveal FcαRI-independent immunomodulatory functions of naturally occurring mIgA and potential therapeutic utility of mIgA in autoimmune and inflammatory diseases that implicate Th17 cells.

## Materials and Methods

### Cell-Culture Reagents and Antibodies

Anti-CD3 (clone UCHT1), anti-CD28 mAbs (clone 37407), and TGF-β1 were procured from R&D Systems (Lille, France). IL-1β, IL-6, and IL-21 were purchased from Immuno Tools (Friesoythe, Germany). Plasma-derived human serum albumin (HSA) was from Laboratoire Française de Biotechnologies (Les Ulis, France).

### Immunoglobulins

Monomeric IgA and F(ab′)_2_ fragments of mIgA and IVIG (Privigen^®^) were provided by CSL Behring AG (Bern, Switzerland).

Monomeric IgA was derived from the AIEX chromatographic step of the IVIG manufacture process of CSL Behring AG. Fraction F4 was obtained after a post-wash of the Macro-Prep High Q (BioRad, Hercule, CA, USA) column with 10 mM phosphate/30 mM acetate at pH 6.5 by elution with 55 mM tartrate/5 mM acetate at pH 7.6. Fraction F4 was then brought to approximately 1 mg/ml in PBS by ultra-/diafiltration and then depleted of IgG by affinity chromatography using an IgSelect resin (GE Healthcare, Glattbrugg, Switzerland). mIgA was directly harvested from the flow through fraction of the IgSelect chromatography and brought to its final formulation *via* ultra-/diafiltration of 48.5 g/l in PBS.

F(ab′)_2_ fragments from IgA were generated by solid phase pepsin digestion using pepsin-coupled beads (Thermo Fisher Scientific, Allschwil, Switzerland). The F(ab′)_2_ fragments were recovered by centrifugation. The supernatant was sterile filtered (0.45 μm) and formulated in PBS using ultrafiltration centrifugal devices (30,000 Da MWCO; Sartorius, Tagelswangen, Switzerland). Purity and integrity were controlled by SDS-PAGE and SE chromatography.

The labeling of mIgA and IVIG was done with the Lightning-Link^®^ Rapid DyLight^®^ 650 kit (Innova Biosciences, Cambridge, UK) according to manufacturer’s instructions.

### Cell Purification

Buffy coats from the healthy donors were processed to purify peripheral blood mononuclear cells (PBMCs). Ethics committee approval for the use of such material (Institut National de la Santé et de la Recherche-EFS ethical committee convention 15/EFS/012) was obtained and experiments were performed in accordance with the approved guidelines of INSERM. The CD4^+^ T cell isolation kit-II (Miltenyi Biotec, Paris, France) was used to isolate untouched total CD4^+^ T cells by negative selection. Subsequently, CD45RA^+^ and CD45RO^+^ CD4^+^ T cells were separated by using CD45RO microbeads (Miltenyi Biotec). Furthermore, CD25^+^ cells were depleted from the CD45RA^+^ fraction by using CD25 microbeads (Miltenyi Biotec) to obtain CD4^+^CD25^−^CD45RO^−^ naïve T cells. The purity of all subpopulations was more than 96%.

Monocytes were isolated from PBMC by using CD14 microbeads (Miltenyi Biotec) and were cultured for 5 days with GM-CSF (1,000 IU/million cells) and IL-4 (500 IU/million cells) (both from Miltenyi Biotec) for the differentiation into DCs (17).

### T-Cell Stimulation and Culture

Forty-eight well flat bottom plates were coated with 1.5 μg/ml anti-CD3 mAb for at least 5 h at 37°C. At the end of incubation, the wells were rinsed once with RPMI-1640 medium. A total of 5 × 10^4^ CD4^+^ T cells/well/500 μl were stimulated in serum-free X-VIVO 15 medium with soluble anti-CD28 mAb (1.0 μg/ml) in presence of cytokines including acid-treated TGF-β1 (5 ng/ml), IL-21 (25 ng/ml) for naïve T cells, and IL-1β (12.5 ng/ml) and IL-6 (25 ng/ml) for memory T cells (18). Indicated concentrations of IgA, F(ab′)_2_ fragments of IgA, IVIG, or HSA were added to the cells 12 h after the initiation of culture. The cells were cultured for 6 days at 37°C in 5% CO_2_. The supernatants were collected at the end of experiments for cytokine analysis and the cells were used for intracellular staining.

### Intracellular Staining for CD4^+^ T Cells and Cytokine Assays

Cells were stimulated with phorbol 12-myristate 13-acetate (50 ng/ml, Sigma-Aldrich, Saint Quentin Fallavier, France) and ionomycin (500 ng/ml, Sigma-Aldrich) at 37°C for 6 h with GolgiStop (BD Biosciences Le Pont de Claix, France) for the last 3 h. Cells were surface stained with BV421-conjugated CD4 mAb (clone RPA-T4, BD Biosciences). Cells were fixed, permeabilized (Fix/Perm, eBioscience, Paris, France), and incubated with APC-conjugated anti-human Foxp3 (clone 236A/E7, eBioscience), PE-conjugated anti-human IL-17A (clone eBio64CAP17, eBioscience), and FITC-conjugated anti-human IFN-γ (Clone 4S.B3, BD Biosciences) at 4°C. Ten thousand cells were acquired for each sample and data were analyzed by using FACS DIVA and Flowjo softwares.

Intracellular staining for the phosphorylated STAT3 (pSTAT3) was carried out on stimulated CD4^+^ T cells at indicated time points. Cells were harvested and fixed in pre-warmed BD Cytofix buffer by incubation for 10 min at 37°C. The cells were washed twice with staining buffer (1% fetal calf serum/PBS), permeabilized with chilled BD Phosflow Perm Buffer III on ice for 30 min. At the end of incubation, the cells were washed twice with staining buffer, incubated with PE-conjugated anti-STAT3 pY705 (clone 4/P-STAT3, BD Biosciences) at room temperature for 45–60 min. Cells were washed and resuspended in staining buffer before acquisition.

Amounts of IL-17A in the cell-free supernatants were quantified by ELISA (DuoSet ELISA kit, R&D Systems).

### Analysis of Expression of CD89 and DC-SIGN

CD4^+^ T cells were stimulated with anti-CD3 and anti-CD28 mAbs for 24 h. Cells were surface stained with BV421-conjugated CD4 mAb and APC-conjugated CD89 (clone A59, BioLegend, London, UK) or FITC-conjugated DC-SIGN (clone DCN46, BD Biosciences) mAbs for 30 min at 4°C. Monocytes were stained with FITC-conjugated HLA-DR (clone TU36, BD Biosciences) and APC-conjugated CD89, while DCs were stained with APC-conjugated HLA-DR (clone G46-6, BD Biosciences) and FITC-conjugated DC-SIGN mAbs. The expression of markers was analyzed by flow cytometry.

### IgA- and IgG (IVIG)-Binding Assay

Total CD4^+^ T cells were stimulated with anti-CD3/CD28 mAbs for 24 hrs. To analyze the binding of IgA or IgG (IVIG) to CD4^+^ T cells, either resting or anti-CD3, anti-CD28-stimulated T cells were incubated with BV421-conjugated anti-human CD4, FITC-conjugated CD45RA (clone HI100, BD Biosciences), and BV510-conjugated CD45RO (clone UCHL1, BD Biosciences) antibodies and DyLight 650-conjugated-IgA or IgG (IVIG) (3 μg/10^6^ cells) for 30 min. Cells were analyzed by flow cytometry.

Immunoglobulin A and IgG binding to IL-1RI and IL-6Rα was measured by ELISA. ELISA plates were coated with recombinant IL-1RI (human IL-1Ra, expressed in 293E cells) and IL-6Rα (human IL-6Rα, expressed in insect cells) (1 μg/ml) (BioLegend) overnight at 4°C. After blocking with 5% BSA in PBS, 0.02% Tween 20 for 1 h at 37°C, wells were incubated with different concentrations of IgA, or IVIG for 2 h at 37°C. Following washings, the plates were incubated with horseradish peroxidase-labeled anti-human IgA or IgG for 2 h, followed by development with 3,3′,5,5′-tetramethylbenzidine. The absorbance was measured at 450 and 570 nm. The data were analyzed by subtracting the absorbance of 570 nm from those of 450 nm.

### Statistical Analysis

The data were analyzed by one-way ANOVA (repeated measures with Tukey’s multiple comparison test) or two-tailed Student’s *t*-test using GraphPad Prism software.

## Results

### mIgA Inhibits Differentiation of Human Th17 Cells

**Figure 1:**
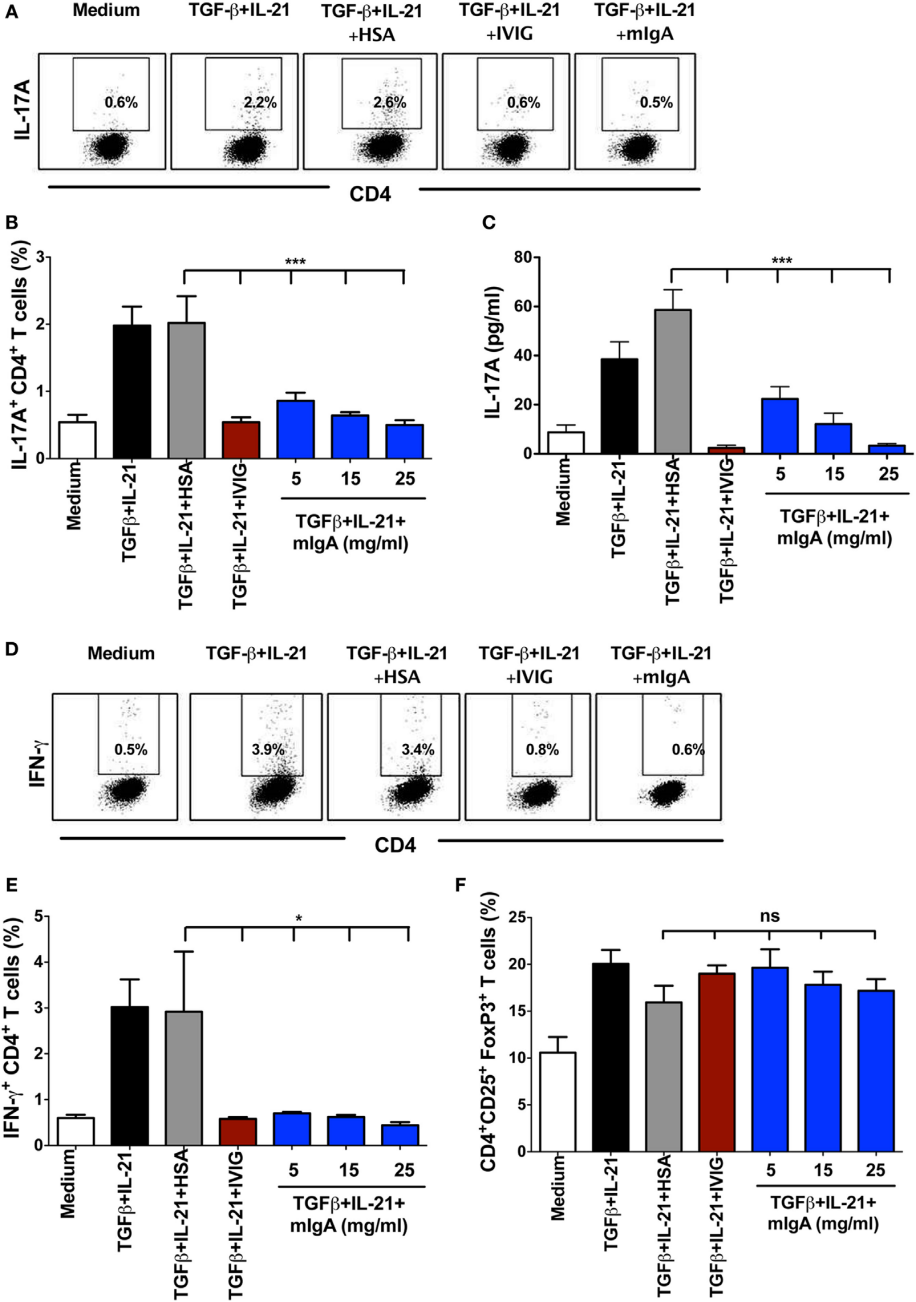
**Monomeric IgA (mIgA) inhibits differentiation of human Th17 cells and affects the generation of IFN-γ^+^CD4^+^ T cells under Th17 differentiation conditions without modulating FoxP3^+^ T cells**. **(A)** Flow cytometry analysis of intracellular IL-17A in the naïve CD4^+^ T cells cultured in serum-free X-vivo medium in the presence of anti-CD3 and anti-CD28 mAbs alone (medium) or stimulated with TGFβ and IL-21 for 6 days. mIgA (25 mg/ml), IVIG (25 mg/ml), or human serum albumin (HSA) (10 mg/ml) (0.15mM) were added to the T cell cultures after 12 h of cytokine stimulation. Data from one of five independent experiments are presented. **(B)** Percentage of IL-17A^+^CD4^+^ T cells (mean ± SEM, *n* = 5 donors) and **(C)** amount of secreted IL-17A (mean ± SEM, *n* = 9 donors) in T cell cultures differentiated under above conditions. mIgA was added at three different concentrations (5, 15, and 25 mg/ml). **(D)** Flow cytometry analysis of intracellular IFN-γ in the naïve CD4^+^ T cells under Th17 differentiation conditions. Data from one of five independent experiments are presented. **(E)** Percentage of IFN-γ^+^CD4^+^ T cells and **(F)** CD4^+^CD25^+^FoxP3^+^ T cells (mean ± SEM, *n* = 5 donors) among CD4^+^ T cells cultured under above conditions. Statistical significance as determined by one-way ANOVA is indicated (*P < 0.05; ***P < 0.001; ns, not significant).

CD45RA^+^CD25^−^ naïve CD4^+^ T cells were stimulated with anti-CD3 and anti-CD28 mAbs in the presence TGF-β and IL-21 for 6 days to differentiate Th17 cells. TGF-β is required to induce both FoxP3 and RORC in naïve T cells and to inhibit IFN-γ while IL-21 relives RORC from the FoxP3 by inducing STAT3 activation (2, 18). Twelve hours after the initiation of culture, mIgA was added to the cells at various concentrations (5, 15, and 25 mg/ml). We observed that mIgA significantly inhibits the differentiation of human Th17 cells (Figures 1A,B) as analyzed by intracellular staining for IL-17A. Significant inhibitory effect was observed even at low concentrations (5 mg/ml) of mIgA. In addition, mIgA also inhibited the production of IL-17A, the signature cytokine of Th17 cells (Figure 1C). The suppressive effect of mIgA was similar to that of therapeutic intravenous immunoglobulin IgG (IVIG) (Figures 1A–C) that is used in the therapy of various autoimmune, inflammatory, and infectious diseases (19–23) and was previously shown to inhibit Th17 responses both in experimental models and in patients with autoimmune diseases (24–29).

The effect of mIgA on the inhibition of Th17 differentiation was specific, as equimolar concentration of HSA (10 mg, 0.15 mM), used as protein control, did not alter Th17 differentiation (Figures 1A–C). Also, the inhibitory effect of mIgA on Th17 responses was not due to toxic effects of the immunoglobulins as we did not observe differences in the yield of cells in mIgA-treated conditions as compared to cytokine-treated control cells or HSA-treated cells.

We also analyzed the effect of mIgA on frequency of IFN-γ-secreting CD4^+^ T cells in the culture. We found that mIgA was equally effective to inhibit IFN-γ responses under Th17 differentiation conditions (Figures 1D,E). Further, similar to our previous report on IVIG (24), inhibition of Th17 differentiation by mIgA was not associated with the reciprocal enhancement of CD4^+^CD25^high^ FoxP3^+^ Tregs (Figure 1F). Thus, these results demonstrate that mIgA exhibits inhibitory effects on the differentiation of human Th17 cells.

### mIgA Suppresses Amplification of Human Th17 Cells

**Figure 2:**
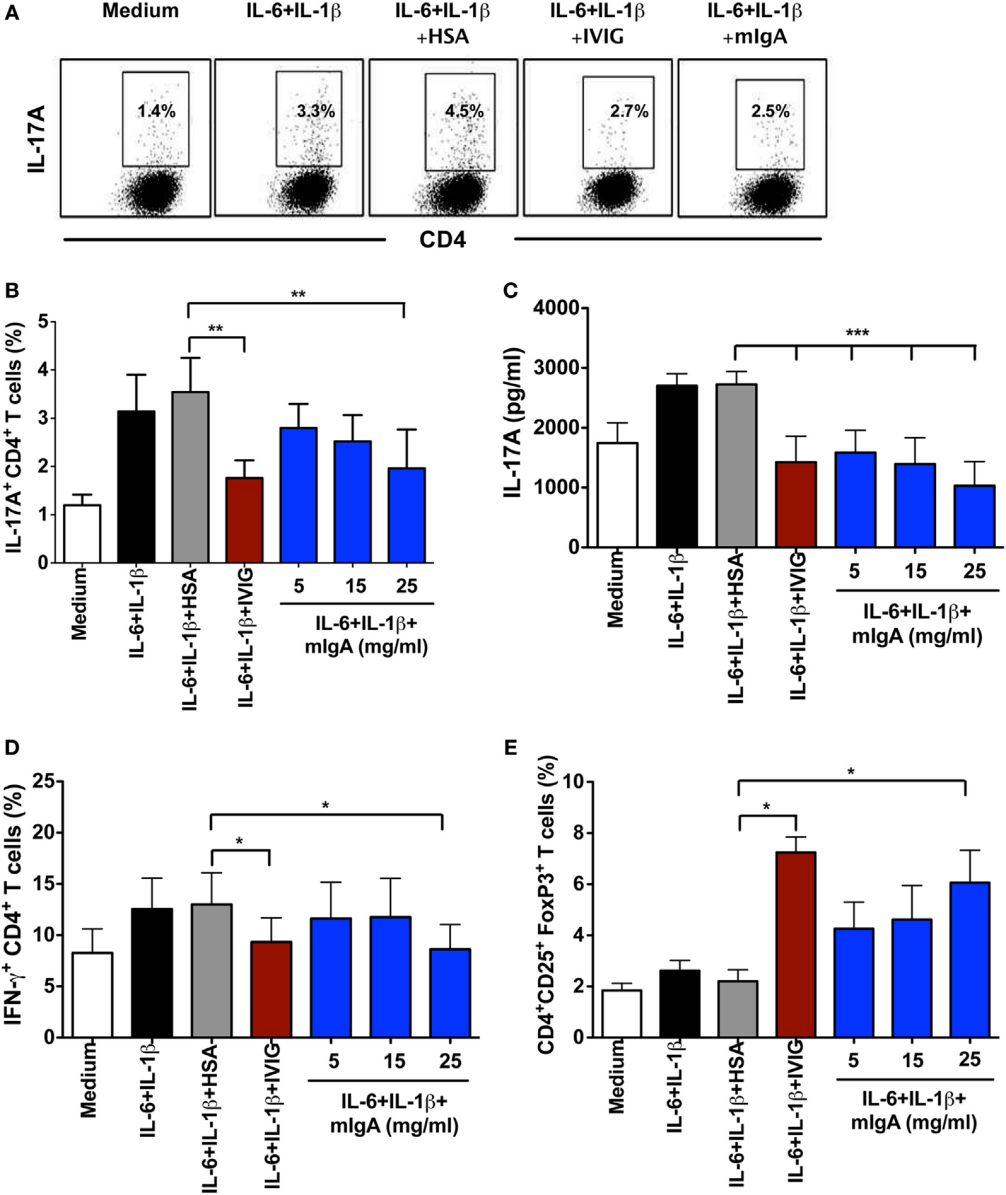
**Monomeric IgA (mIgA) reciprocally regulates Th17 cells and FoxP3^+^ T cells under Th17 amplification conditions**. **(A)** Flow cytometry analysis of intracellular IL-17A in the memory CD4^+^ T cells cultured in serum-free X-vivo medium in the presence of anti-CD3 and anti-CD28 mAbs alone (medium) or stimulated with IL-6 and IL-1β for 6 days. mIgA (25 mg/ml), IVIG (25 mg/ml), or human serum albumin (HSA) (10 mg/ml) (0.15mM) were added to the T cell cultures after 12 h of cytokine stimulation. Data from one of five independent experiments are presented. **(B)** Percentage of IL-17A^+^CD4^+^ T cells (mean ± SEM, *n* = 5 donors) and **(C)** amount of secreted IL-17A (mean ± SEM, *n* = 9 donors) in T cells cultured under above conditions. mIgA was added at three different concentrations (5, 15, and 25 mg/ml). **(D)** Percentage of IFN-γ^+^CD4^+^ T cells (mean ± SEM, *n* = 5 donors) and **(E)** CD4^+^CD25^+^FoxP3^+^ T cells (mean ± SEM, *n* = 5 donors) among CD4^+^ T cells cultured under above conditions. Statistical significance as determined by one-way ANOVA is indicated (*P < 0.05, **P < 0.01, ***P < 0.001).

For the amplification of human Th17 cells, CD45RO^+^ memory CD4^+^ T cells were stimulated with anti-CD3 and anti-CD28 mAbs in the presence of IL-1β and IL-6 for 6 days. By signaling through corresponding cytokine receptors, IL-1β and IL-6 induce phosphorylation of STAT3 and amplify Th17 cells in the memory CD4^+^ T cell pool (2, 18). Similar to Th17 cell differentiation conditions, various concentrations of mIgA (5, 15, and 25 mg/ml) were added to the cells 12 h post initiation of culture. The pro-inflammatory cytokines IL-1β and IL-6 significantly enhanced the frequency of IL-17A-producing cells as compared to memory CD4^+^ T cells stimulated with anti-CD3 and anti-CD28 mAbs alone (Figures 2A,B). Remarkably, mIgA significantly suppressed the amplification of Th17 cells (Figures 2A,B) and the amount of IL-17A (Figure 2C) produced by these cells. Its inhibitory effect was similar to that of high-dose IVIG (Figures 2A–C). Of note, the inhibitory effect of mIgA on IFN-γ responses under Th17 amplification conditions was observed only at the highest immunoglobulin concentration (Figure 2D).

Under Th17 amplification conditions, the inhibitory effect of mIgA on Th17 cells was associated with a reciprocal enhancement of FoxP3^+^ Tregs (Figure 2E). The effect of mIgA on Tregs was prominent at the highest concentration (25 mg/ml). These results thus suggest that both mIgA and IVIG exert similar modulatory effects on Th17 amplification.

### FcαRI (CD89) and DC-SIGN Are Dispensable for the Inhibition of Th17 Response by mIgA

**Figure 3:**
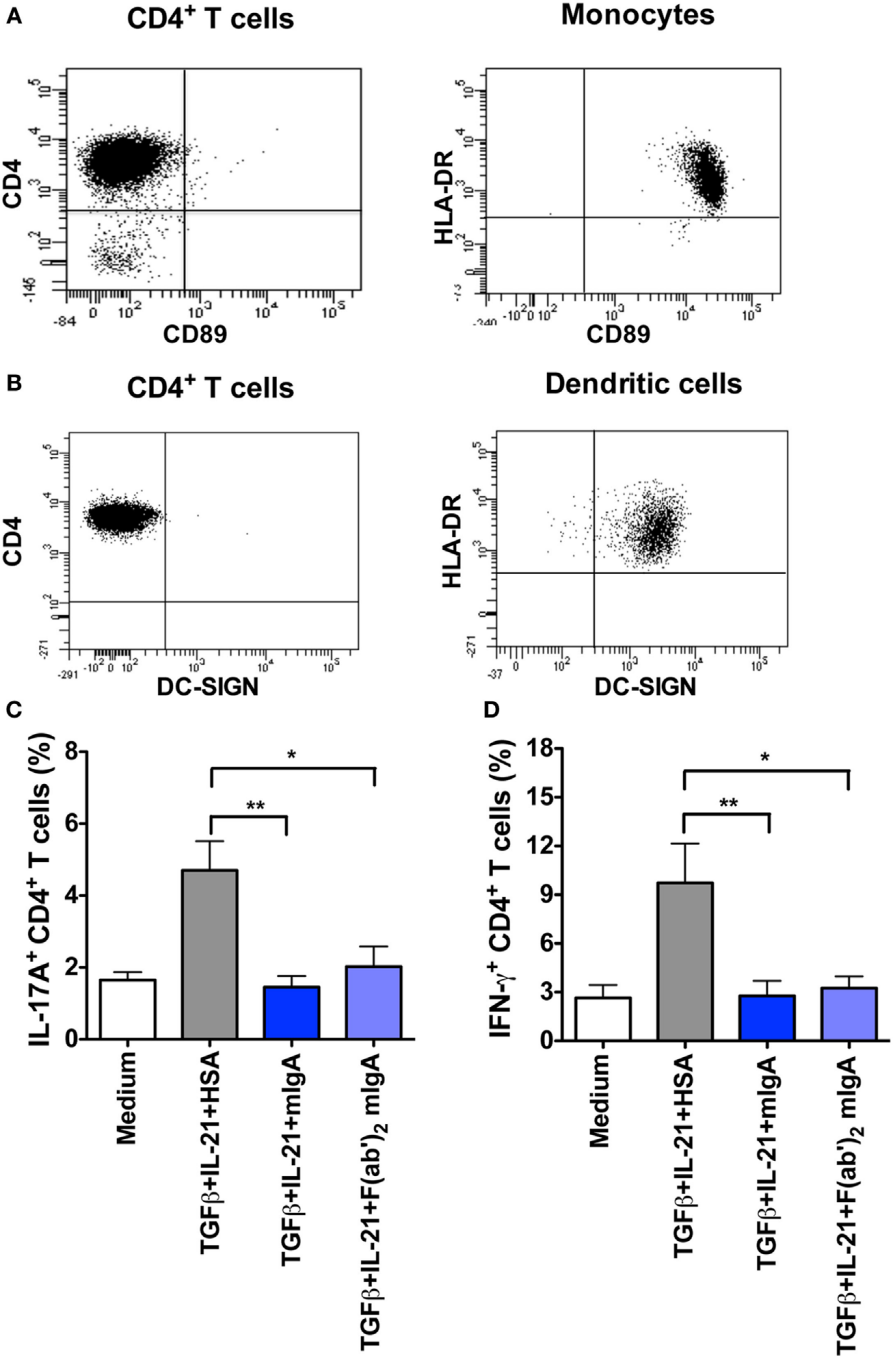
**Inhibition of Th17 response by monomeric IgA (mIgA) implicates F(ab′)_2_ fragments while CD89 and DC-SIGN are dispensable**. **(A,B)** Flow cytometric analysis human CD4^+^ T cells, monocytes, and dendritic cells for the expression of CD89 and DC-SIGN. **(C,D)** Percentage of **(C)** IL-17A^+^CD4^+^ T cells and **(D)** IFN-γ^+^CD4^+^ T cells (mean ± SEM, *n* = 4 donors) in T cells cultured in serum-free X-vivo medium in the presence of anti-CD3 and anti-CD28 mAbs alone (Medium) or stimulated with TGFβ and IL-21 for 6 days. mIgA (25 mg/ml), F(ab′)_2_ fragments of mIgA (15 mg/ml), or human serum albumin (HSA) (10 mg/ml) (0.15mM) were added to the T cell cultures after 12 h of cytokine stimulation. Statistical significance as determined by one-way ANOVA is indicated (*P < 0.05; **P < 0.01).

Recent reports have indicated that FcαRI (CD89) plays a major role in mediating anti-inflammatory effects of mIgA on innate immune cells (10–13, 16). In addition, SIGN-R1 on DCs was also implicated in the immunoregulatory functions of secretory IgA (30). To explore if FcαRI and DC-SIGN (human counterpart of SIGN-R1) receptors are implicated in the inhibitory effect of mIgA, we investigated the expression of these two receptors on activated CD4^+^ T cells. We found that human CD4^+^ T cells were negative for both, FcαRI and DC-SIGN (Figures 3A,B), thus ruling out their implication in the inhibition of Th17 responses by mIgA. Importantly, monocytes and DCs that were used as positive controls stained with specific anti-FcαRI and anti-DC-SIGN fluorescent antibodies, respectively (Figures 3A,B).

### Inhibition of Th17 Response by mIgA Implicates F(ab′)_2_ Fragments

Lack of expression of FcαRI and DC-SIGN on CD4^+^ T cells raised an important prospect that suppressive effects of mIgA on Th17 cell responses might be mediated *via* F(ab′)_2_ fragments. Indeed, we found that F(ab′)_2_ fragments of mIgA significantly inhibited the frequency of IL-17A-positive T cells under Th17 differentiation conditions (Figure 3C). This effect was also associated with significant downregulation of IFN-γ-secreting CD4^+^T cells in the culture (Figure 3D).

### mIgA Binds to CD4^+^ T Cells

**Figure 4:**
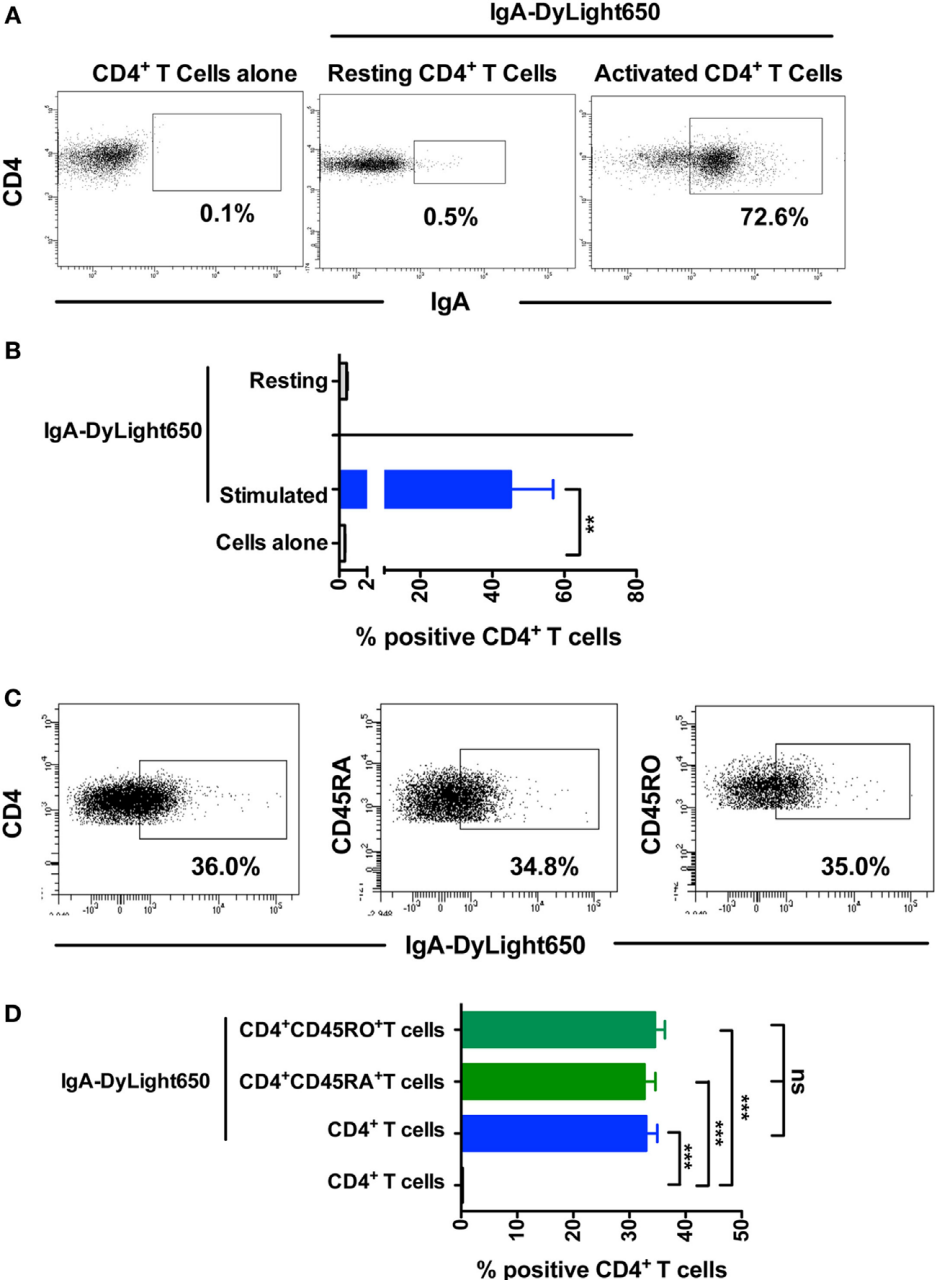
**Monomeric IgA (mIgA) binds to CD4^+^ T cells**. **(A,B)** Representative dot plots and percentage (mean ± SEM, *n* = 3–9 donors) of binding of DyLight650-conjugated mIgA to CD4^+^ T cells. Statistical significance as determined by two-tailed Student’s *t*-test is indicated (**P < 0.01). **(C,D)** Representative dot plots and percentage (mean ± SEM, *n* = 4) of binding of DyLight650-conjugated mIgA to CD4^+^ T cells, CD4^+^CD45RA^+^ naïve T cells, and CD4^+^CD45RO^+^ memory T cells. Statistical significance as determined by one-way ANOVA is indicated (**P < 0.01; ***P < 0.001; ns, not significant).

Natural antibodies that recognize various self-motifs have been identified (31–34). Therefore, to further understand the mechanisms underlying the inhibitory effect of IgA on Th17 responses, we analyzed the binding of mIgA to CD4^+^ T cells by flow cytometry. We found that nearly 45% of the stimulated CD4^+^ T cells (*n* = 9 donors) were positive for mIgA binding (Figures 4A,B). mIgA however did not bind to resting CD4^+^ T cells (*n* = 3 donors) suggesting that activation signals license T cells for immunoglobulin binding. Further, the extent of binding of mIgA was similar for CD45RA^+^ naïve and CD45RO^+^ memory CD4^+^ T cells (Figures 4C,D). This finding indicates that naturally occurring mIgA exerts its immunomodulatory effects by binding to CD4^+^ T cells.

### mIgA and IVIG Recognize CD4^+^ T Cells to a Similar Extent

**Figure 5:**
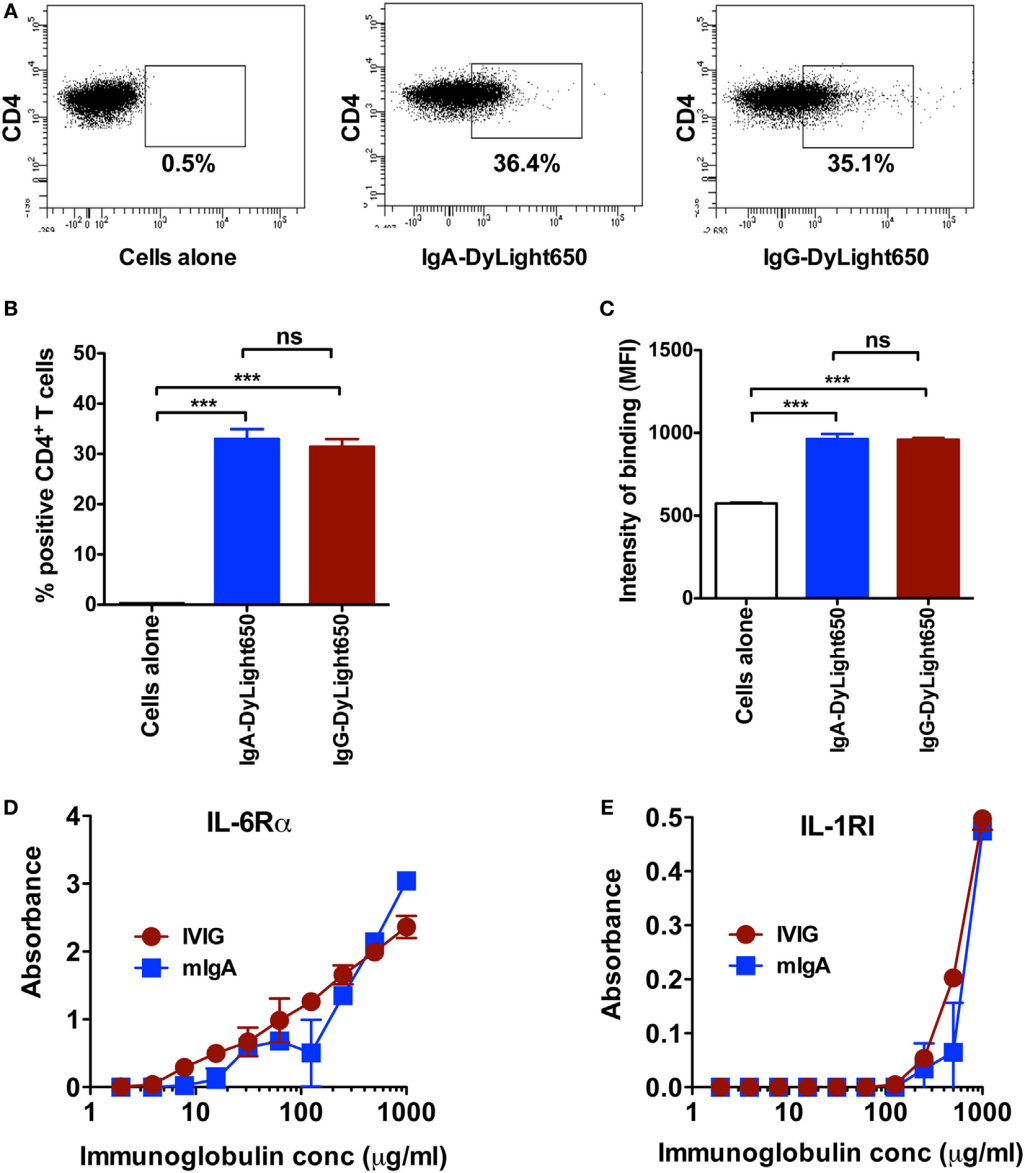
**Monomeric IgA (mIgA) and IVIG recognize CD4^+^ T cells and cytokine receptors to a similar extent**. **(A–C)** Representative dot plots, percentage (mean ± SEM, *n* = 4), and intensity (mean ± SEM, *n* = 4) of binding [as shown by median fluorescence intensity (MFI)] of DyLight650-conjugated mIgA and IVIG to CD4^+^ T cells. **(D,E)** Binding of mIgA and IVIG to recombinant IL-6Rα and IL-1RI as analyzed by ELISA. Immunoglobulins were tested at serial concentrations (1–0.002 mg/ml). Statistical significance as determined by one-way ANOVA is indicated (***P < 0.001; ns, not significant).

As both mIgA and IVIG reciprocally regulated Th17 and Tregs at equivalent concentration, rises the possibility that they recognize CD4^+^ T cells to a similar extent. Confirming our proposition, we observed that both immunoglobulin fractions bind CD4^+^ T cells to a similar magnitude (Figures 5A–C). To further substantiate these results, we investigated the surface molecules on CD4^+^ T cells that could be recognized by mIgA and IVIG. As mIgA and IVIG inhibited cytokine-mediated Th17 differentiation and amplification, we hypothesized that the corresponding cytokine receptors on CD4^+^ T cells are the targets for these immunoglobulin fractions. Therefore, we analyzed binding of mIgA and IVIG to IL-6Rα and IL-1RI, the receptors for IL-6 and IL-1β that are implicated in the amplification of Th17 cells (1, 2, 18, 35). mIgA recognized both IL-6Rα and IL-1RI in a dose-dependent manner (Figures 5D,E). The binding of mIgA was however stronger for IL-6Rα. Importantly, IVIG and mIgA showed similar pattern of recognition of IL-6Rα and IL-1RI (Figures 5D,E).

### mIgA Interferes with STAT3 Activation

**Figure 6:**
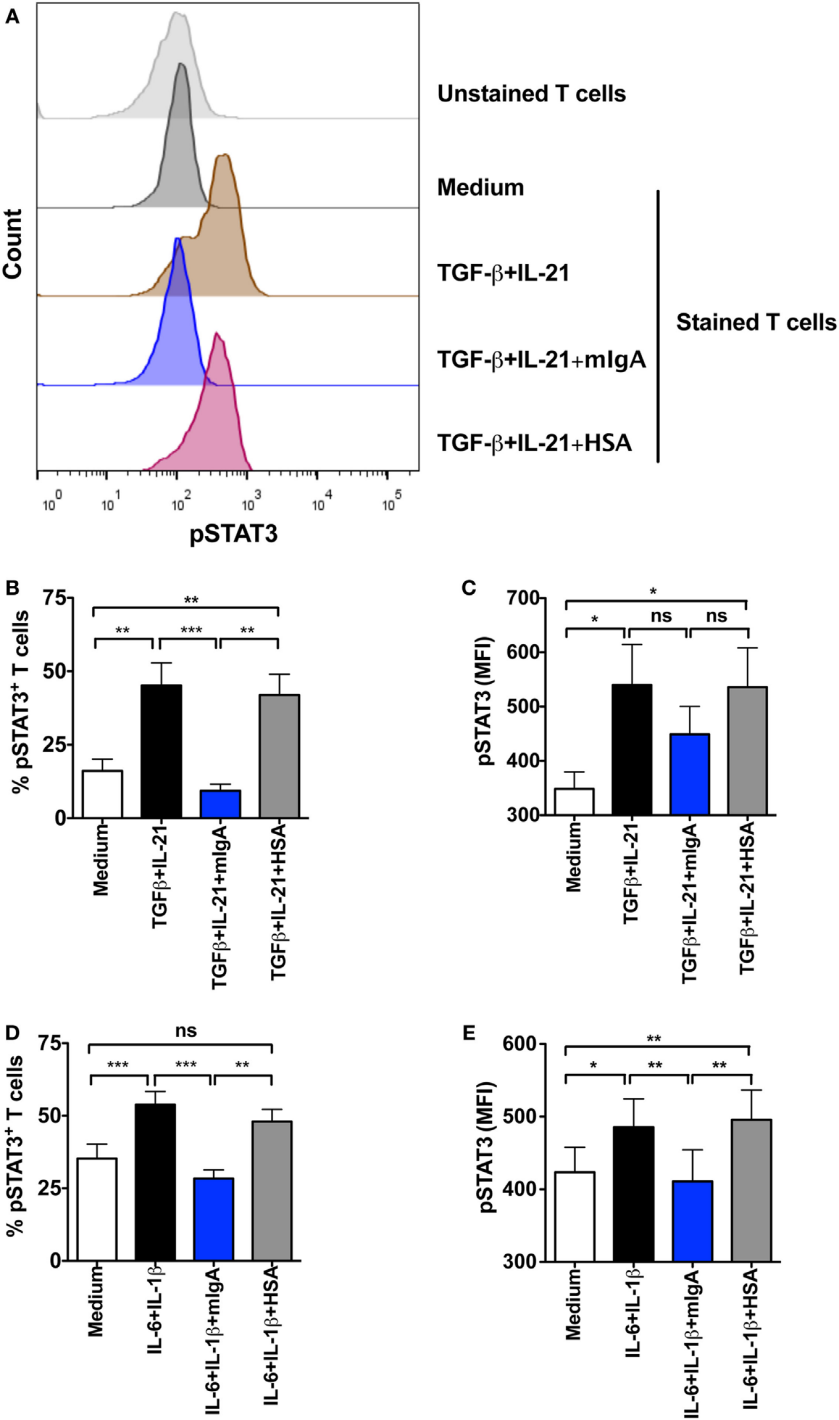
**Monomeric IgA (mIgA) interferes with signal transducer and activator of transcription 3 (STAT3) activation**. CD4^+^ T cells were cultured in serum-free X-vivo medium in the presence of anti-CD3 and anti-CD28 mAbs alone (medium) or stimulated with cytokines for the differentiation of Th17 cells [TGFβ and IL-21, panels **(A–C)**] or for the amplification of Th17 cells [IL-1β and IL-6, panels **(D,E)**]. mIgA (25 mg/ml) or human serum albumin (HSA) (10 mg/ml) (0.15 mM) were added to the T cell cultures. Phosphorylation of STAT3 was analyzed after 72 h. **(A)** Representative histograms showing pSTAT3 in different experimental conditions for differentiating Th17 cells. **(B–E)** Percentage of pSTAT3^+^CD4^+^ T cells **(B,D)** and median fluorescence intensity (MFI) of pSTAT3 **(C,E)** under Th17 differentiation **(B,C)** (mean ± SEM, *n* = 5 donors) and amplification **(D,E)** (mean ± SEM, *n* = 5 donors) conditions. Statistical significance as determined by one-way ANOVA is indicated (*P < 0.05; **P < 0.01; ***P < 0.001; ns, not significant).

STAT3 has a key role in the Th17 cell programming by relieving RORC from FoxP3-mediated inhibition. Together with RORC, it facilitates the secretion of effector cytokines of Th17 cells. STAT3 is activated by Th17-polarizing cytokines (1, 2). As we found that mIgA recognizes cytokine receptors implicated in Th17 responses, we aimed at exploring if this binding of mIgA on CD4^+^ T cells has a repercussion on STAT3 phosphorylation and hence interferes with early signaling events of Th17 cells. In line with our proposition, we uncovered that mIgA significantly suppresses the phosphorylation of STAT3 at Y705 both in Th17 differentiation and amplification conditions (Figures 6A–E).

## Discussion

Patients with selective IgA deficiency not only exhibit enhanced predisposition to mucosal infections but also to several autoimmune and allergic conditions including arthritis, autoimmune endocrinopathies, and intestinal inflammatory diseases such as ulcerative colitis and Crohn’s disease (36). These observations support the role of IgA in the immune regulation and homeostasis. The immunoregulatory functions of IgA are mediated mainly *via* FcαRI, expressed on various innate immune cells. Whether such an interaction is inflammatory or anti-inflammatory is determined by nature of the IgA. Thus, IgA immune complexes and polymeric IgA were reported to induce activation of innate immune cells while mIgA was found to be anti-inflammatory. The anti-inflammatory effects of mIgA include its inhibitory effect on the chemotaxis of immune cells, IgG-mediated phagocytosis and bactericidal activity of polymorphonuclear cells, and secretion of inflammatory cytokines such as TNF and IL-6 (37–43). Furthermore, mIgA also induces the production of IL-10 by human monocytes and monocyte-derived DCs (44). Of note, recognition of secretory IgA *via* carbohydrate-recognizing receptors on innate cells such as SIGN-R1 has also been suggested (30, 45, 46). This interaction rendered DC tolerogenic characterized by the secretion of IL-10 and gaining the ability to expand Tregs. Our current results show that mIgA exerts direct anti-inflammatory functions on effector T cells, independent of FcαRI, DC-SIGN, and innate cells. These data thus further expand the landscape of immunoregulatory functions of IgA and of natural immunoglobulins.

Aberrant activation of Th17 cells and their effector cytokines IL-17A and GM-CSF are implicated in the pathogenesis of various autoimmune and inflammatory diseases such as systemic lupus erythematosus, rheumatoid arthritis, psoriasis, dermatomyositis, allergy, asthma, and others (2). Importantly, targeting Th17 responses have given promising results in experimental models of autoimmune diseases and in patients (47). Recent data from ours and others show that beneficial effects of therapeutic IVIG containing IgG from pooled plasma of thousands of healthy donors is associated with inhibition of Th17 responses (24–29, 48–50), indicating that immunoglobulins have regulatory functions on Th17 cells. The data from current report show that in addition to IgG, mIgA also exerts modulatory effects on Th17 responses. In fact, mIgA was recently demonstrated to attenuate experimental arthritis in human CD89 transgenic mice (11), a disease where Th17 cells have a key role in the pathogenesis. It should be noted that the serum levels of IgA ranges from 2 to 3 mg/ml, but we observed consistent inhibitory effect of mIgA both on differentiation and amplification of Th17 cells at higher doses (25 mg) and was analogous to what is observed with IVIG (24). The lower concentration of mIgA (5 mg) although shown inhibitory effects on Th17 differentiation, significant effects were not observed on all parameters of Th17 amplification. Immunoglobulins exert their anti-inflammatory effects *via* several mutually non-excusive mechanisms and it might explain requisite of higher concentrations of immunoglobulins for the therapeutic purposes to inhibit inflammation.

Recent reports show that Th17 cells are required for the production of high affinity secretory IgA at intestinal mucosal surfaces (51, 52). Payer patch-homing Th17 cells induce IgA-producing germinal center B cells by acquiring the phenotype of follicular T cells and producing IL-21. A similar mechanism was also reported for the promotion of local IgA responses in lungs by vaccine-induced Th17 cells (53). Thus, Th17 cells and secretory IgA work in cooperation to protect mucosal surfaces against microbial invasion and regulating the microbiota. Although Th17 cells were reported to provide help for B cells to produce systemic IgG response *via* IL-21 and IL-17 (54), their role for circulating IgA response is not known. As we uncovered that mIgA inhibits Th17 responses, these data together suggest that under the conditions where Th17 cells are hyperactivated, IgA has the ability to control its own helpers to keep the immune response at check.

Natural antibodies (immunoglobulins) that are produced in the absence of deliberate immunization and independent of external antigens constitute an integral part of immunoglobulin repertoire (55–64). A major fraction of these natural antibodies recognize self-motifs and are termed as natural autoantibodies. These natural autoantibodies have important role in the therapeutic benefits of IVIG in autoimmune and inflammatory conditions (23, 31, 65). Our results indicate that inhibitory effect of mIgA on Th17 cells is mediated in part *via* natural IgA autoantibodies that recognize cytokine receptors on CD4^+^ T cells and interfere with Th17 programming. In fact, we found that both mIgA and IVIG recognize CD4^+^ T cells as well as IL-6Rα and IL-1RI to a similar extent. Also, significant downregulation of STAT3 phosphorylation by mIgA support our proposition.

Although we found that mIgA directly inhibits Th17 responses independent of FcαRI and DC-SIGN, we believe that the effect of mIgA on Th17 cells *in vivo* also implicates innate cells such as DCs, monocytes, and macrophages, which are known to provide signals for Th17 responses (2). In fact, several reports have now shown that mIgA exerts FcαRI-mediated anti-inflammatory effects on innate cells by prompting ITAMi configuration (10–13, 16). It should be noted that IVIG (IgG) and IgA display distinct differences in their glycosylation pattern. IgG is glycosylated at Asn297 of the Fc-fragment and about 15–25% of IgG are glycosylated at the Fab region (66). IgA on the other hand is the most glycosylated form of immunoglobulin and over 6% of IgA content are represented by sugars (67). In addition to *N*-linked glycans at asparagine 263 and asparagine 459 of Fc region, up to five *O*-linked glycan chains containing of *N*-acetylgalactosamine with β1,3-linked galactose and sialic acids can be found at the hinge region serine and threonine residues of IgA1, the predominant IgA subclass in the circulation (68, 69). Similar to IgG, about 30% of Fab fragments of IgA1 also contain *N*-linked glycans (69). Further, IgA1 and IgG display significant differences in the sialylation content of *N*-glycans. In contrast to IgG that contains sialic acid in less than 10% *N*-glycans, nearly 90% of the *N*-glycans in IgA1 are sialylated mainly with α2,6-configuration (69). Whether differences in the glycosylation patterns of IgA and IgG impact modulation of innate cells and innate cell-mediated Th17 responses remains to be investigated. Due to heterogeneous composition of glycan chains, further work is also necessary with mIgA preparations containing defined glycosylation patterns to finely dissect the role of IgA in modulating Th17 responses. Since F(ab′)_2_ fragments of mIgA could inhibit Th17 responses similar to intact mIgA and that F(ab′)_2_ fragments of IVIG were previously reported to inhibit Th17 responses both *in vitro* and *in vivo* (24, 70) imply that *N*-linked glycans (and hence sialylation) at Fc region of mIgA might not have a role in regulating Th17 responses.

In conclusion, our data highlight the promise of plasma-derived mIgA as therapeutic molecule for autoimmune and inflammatory diseases and hence represents an innovative plasma-derived therapeutic product (71). Although shorter half-life of IgA as compared to IgG is the major drawback, the efficacy of mIgA on Th17 cells should instigate the therapeutic development of monomeric plasma IgA as an analog to IVIG.

## Author Contributions

SK and JB designed the research. CS, MD, VP, ES-V, and MS performed the research. SW, MJ, and CV provided research tools. CS, MD, VP, SW, MJ, CV, SK, and JB contributed to data analyses and data interpretation. JB wrote the manuscript. CS, MD, VP, ES-V, MS, SW, MJ, CV, SK, and JB revised the manuscript critically for important intellectual content and approved the final version.

## Conflict of Interest Statement

SW, MJ, and CV are employees of CSL Behring. The reviewer LR declared a shared affiliation and a past coauthorship with several of the authors (JB and SK) to the handling editor, who ensured that the process met the standards of a fair and objective review. The remaining authors declare that the research was conducted in the absence of any commercial or financial relationships that could be construed as a potential conflict of interest.
